# Unexpected Strong Polygyny in the Brown-Throated Three-Toed Sloth

**DOI:** 10.1371/journal.pone.0051389

**Published:** 2012-12-19

**Authors:** Jonathan N. Pauli, M. Zachariah Peery

**Affiliations:** 1 Department of Forest and Wildlife Ecology, University of Wisconsin-Madison, Madison, Wisconsin, United States of America; Université de Sherbrooke, Canada

## Abstract

Promiscuous mating strategies are much more common than previously appreciated. So much so, that several authors have proposed that promiscuity is the “rule” rather than the exception in vertebrate mating systems. Decreasing species mobility and increasing habitat fragmentation have both been suggested to reduce the “polygyny potential” of the environment and promote other mating strategies like promiscuity in females. We explored the social and genetic mating system for one of the most sedentary extant mammals, the brown-throated three-toed sloth (*Bradypus variegatus*), within a highly fragmented Neotropical habitat. Surprisingly, we found that three-toed sloths were strongly polygynous, with males excluding male competitors from their core ranges, and exhibiting strong reproductive skew. Indeed, only 25% of all resident adult males sired offspring and one individual sired half of all sampled juveniles. Paradoxically, a sedentary life-history strategy seems to facilitate polygyny in fragmented landscapes because multiple females can persist within small patches of habitat, and be monopolized by a single male. Our work demonstrates that strong polygyny can arise in systems in which the polygyny potential should be extremely low, and other strategies, including promiscuity, would be favoured. Mating systems can be influenced by a multitude of factor and are dynamic, varying among taxa, over time, and across habitats; consequently, mating systems remain difficult to predict based on general ecological principles.

## Introduction

Enormous progress has been made describing the diversity of mating systems and exploring the ecological and evolutionary mechanisms that shape them. Until recently, it was widely held that passerine birds were principally monogamous and that polygyny predominated among mammals [1]. With the advent of molecular techniques, however, we have realized that genetic mating systems often do not match observed social ones. In particular, female promiscuity is more common than previously recognized, even in socially monogamous or polygynous species [2], [3], [4]. Indeed, nearly 90% of passerine species [5], and >130 species of surveyed mammals [6] exhibit some level of female promiscuity. Even the mating strategies of several mammalian species that were considered exemplars of strict polygyny (e.g., white-tailed deer, *Odocoileus virginianus* [7], roe deer, *Capreolus capreolus* [8]) or monogamy (North American beaver, *Castor candensis* [9], common mole rats, *Crptomys hottentotus* [10]) have been questioned because of high rates of female promiscuity. Such findings have led some to suggest that promiscuous mating systems are the rule rather than the exception, at least in mammals [11], [12].

Promiscuity is believed to have evolved in mammals primarily as a strategy by which females obscure paternity and thereby reduce the risk of infanticide, although a variety of alternative explanations such as increasing the genetic fitness of offspring have been proposed [6]. Promiscuity seems to be a favourable strategy when species occur in highly fragmented habitats and competition among males for mates is enhanced [13]. Moreover, promiscuity is expected in species with limited mobility because males are unable to monopolize access to mating opportunities with females [14]. Thus, both habitat fragmentation and limited mobility reduce the “polygyny potential” of the environment [15] and would seem to reinforce female promiscuity. Nevertheless, making predictions about what mating system a particular vertebrate species will possess has proven challenging [16]. Indeed, the sheer diversity of mating strategies that have been characterized suggests that a syndrome of species-specific characters and environmental factors may ultimately shape mating systems. Mating systems can be flexible across time [17], degree of sociality [12], and resource availability [11].

Three-toed sloths (*Bradypus* spp.) are among the most sessile species of all the known vertebrates. Their extremely low metabolic rates, <75% of that predicted based on body size, and low-quality folivorous diet dictate ≥14 hours of inactivity daily [18]. Indeed, three-toed sloths descend to the forest floor only a handful of times per month to defecate, before reassuming their position in the canopy to forage [19]. Despite being a charismatic and common species in parts of the Neotropics, the mating system of three-toed sloths is virtually unknown. In a study of a related species, Peery and Pauli [14] found that the mating system of Hoffmann’s two-toed sloth (*Choloepus hoffmanni*) involved a mixture of weak polygyny and female promiscuity. Being even more sedentary than two-toed sloths, three-toed sloths provide an opportunity to test the effect of limited mobility on mating tactics in a species at the extreme end of the spectrum of vertebrate life histories.

Herein, we describe the mating and social system of brown-throated three-toed sloths (*Bradypus variegatus*) in a fragmented Neotropical landscape in northeastern Costa Rica. We hypothesized that the extreme sedentary lifestyle of three-toed sloths coupled with a fragmented landscape would erode the polygyny potential for males and select for increased promiscuity among females. Specifically, we predicted that sloths would exhibit weak or no territoriality or mate guarding and exhibit low reproductive skew among males. We predicted that low dispersal power would result in the spatial clustering of close relatives which, in turn, could promote tolerance among neighbouring males, as was observed in Hoffmann’s two-toed sloths [14]. To test these hypotheses, we conducted paternity analyses for juveniles sampled with their putative mothers, quantified spatial overlap among adults using radio-telemetry, and estimated genetic relatedness of co-occurring individuals.

## Methods

### Ethics Statement

All sloths captured, handled, and tracked as part of this study were conducted as stipulated and authorized by IACUC protocol A01424 by the University of Wisconsin-Madison, and adhered to the guidelines for the use of mammals in research set forth by the American Society of Mammalogists [20]. All necessary permits were obtained for the described field studies. Permission to conduct fieldwork was granted by the private landowner, our project and sample collection was approved by the Ministerio de Ambiente, Energia y Telecomunicaciones, Sistema Nacional de Áreas de Conservación, Costa Rica and all samples were imported to the United States with CITES and United States Fish and Wildlife Service approval.

### Study Area and Species

**Figure 1:**
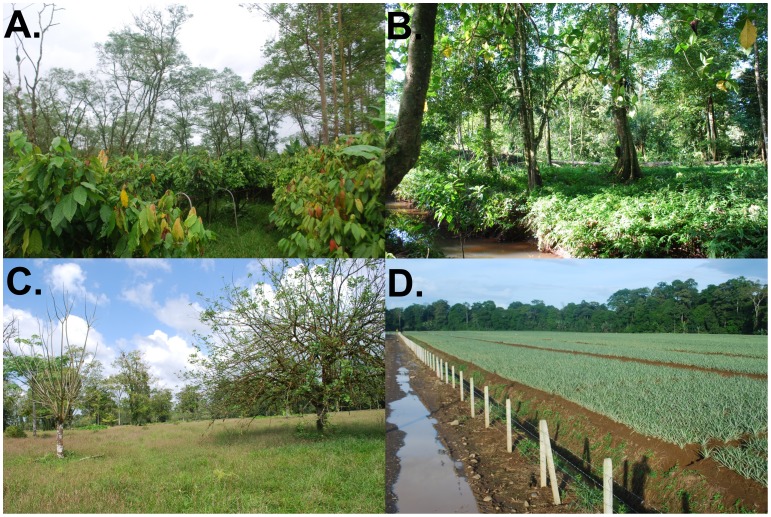
Four dominant cover types present at our study site in northeastern Costa Rica, 2010–2012. **A.** shade grown cacao (*Theobroma cacao*) plantation; **B.** intact strips of tropical rainforest along riparian corridors; **C.** cattle pasture and; **D.** monocultures of pineapple (*Ananas comosus*; shown) and banana (*Musa* spp).

Fieldwork was conducted in a highly fragmented landscape centered on a shade-grown cacao (*Theobroma cacao*) farm (10.32°N, −83.59°W) located 85 km northeast of San José, Costa Rica within the Premontane Humid Life Zone [21]. The study area contained three general habitat types: a shade-grown cacao plantation consisting of cacao trees 3–4 m in height planted underneath a diverse overstory of native shade trees, tropical forest occurring in narrow (∼20 m) riparian buffers, and cattle pasture, and was bordered by monocultures of banana (*Musa* spp.) and pineapple (*Ananas comosus*) plantations (Fig. 1). Three-toed sloths are regularly observed along the living fence rows of the cacao farm as well as along the riparian forests, foraging during the day on the leaves of trees. Females bear a single young each breeding event [22], and are dependent on their mother for ∼5 months. The timing of breeding events are irregular, but seem to generally occur before or during on the rainy season [22]; in our study area, young are often born between November and December, but have also been observed in April and May.

### Captures and Radio-Tracking

We captured sloths by hand from trees from February, 2010 through May, 2012. We determined the sex of each adult sloth from their external genitalia and classified individuals into one of three stage classes (juvenile, subadult, or adult). A sloth was considered to be a juvenile if it was held by or located immediately adjacent (i.e., the same tree) to its putative mother and a subadult once it was no longer observed in direct proximity to its mother. Females were considered to be adults if their body mass was ≥3.7 kg because all captured females known to be at least three years of age (based on previous captures by Vaughan et al. [23]) weighed at least 3.7 kg (*n* = 5) and age of sexual maturity has been estimated to be 2–3 years for both sexes in maned sloths (*Bradypus torquatus*) [24]. Moreover, the smallest female captured with a juvenile weighed 3.7 kg (*n* = 11). Males were considered adults if they weighed at least 3.8 kg, which was equal to the body mass of the smallest male known to be at least three years old [23], and similar to the smallest male (4.2 kg) that was assigned paternity with >95% confidence.

We marked captured individuals with uniquely coded PIT tags (Biomark, Boise, ID) inserted subcutaneously between the shoulder blades. Male and female sloths of adequate size were fitted with radio-collars (Mod-210, Telonics Inc., Mesa, AZ) or uniquely identifiable colour necklaces. Juveniles and small subadults were marked with temporary paint (All-Weather®, Elk Grove, IL) on their backs. Because paint markings were not permanent or unique, we identified these smaller-bodied individuals by capturing them and scanning their PIT tags. We relocated marked sloths 5 to 6 times per week.

### Quantifying Home Range and Spatial Overlap

We estimated home ranges for 30 of the 40 adult sloths with using 90% fixed kernel methods [25]: two radio-collared sloths died of natural predation and were observed ≤6×, seven radio-collared individuals dispersed (i.e., exhibited progressive unidirectional movement away from their original home range), and one sloth with a colour necklace was observed 3× before disappearing from the study site. The remaining adult sloths used in home range estimation were relocated a median of 48 times (range: 16–150) from February, 2010 through May, 2012. Home range analyses were conducted in ArcGIS 10.0 and the Geospatial Modelling Environment using least squares cross validation (LSCV) as the smoothing parameter for each individual.

We quantified home range overlap between all pairs of adults by calculating the utilization distribution overlap index (UDOI) [26] with the adehabitat package [27] in the R statistical environment [28]. The UDOI is based on the product of the two home range utilization distributions and generally ranges from 0 (no overlap) to 1 (100% overlap), although the index can be >1 when utilization distributions are non-uniformly distributed and exhibit a high-degree of overlap [26].

### Kinship Analyses

We sampled sloths via a skin biopsy (∼25 mg) and genotyped sampled individuals at 13 microsatellite markers (B11, D101, B107, D102, D106, D110, B111, D113, D112, D119, C5, B102, and B124), developed specifically for brown-throated three-toed sloths [29]. All loci were unlinked and did not deviate from expectations under Hardy-Weinberg equilibrium. We extracted genomic DNA from tissue samples using the DNeasy extraction kit (Qiagen, Valencia, CA), which yielded 10–25 ng/µL of DNA. PCR reactions were performed in 10 µL volumes containing the following ingredients: 2 ng/µL DNA, 0.8 mM dNTPs, 2 mM MgCl_2_, 0.6 µM of each primer, 0.3 U Taq DNA polymerase (Qiagen), and 0.4 µg/µL BSA. PCR conditions varied by primer but all consisted of the following three steps: (1) 3 min denaturing at 94°C, (2) 25–35 cycles of 40 s at 94°C, 40 s at 55–63°C, and 30 s at 72°C, and (3) a final extension of 4 min at 72°C. Primers were labelled with one of the following fluorescent dyes: HEX, FAM (University of Wisconsin Biotechnology Center, Madison, WI), or NED (Applied Biosystems) and microsatellite sizes were estimated using the software GeneMapper v4.5 (Applied Biosystems). We were able to resolve greater than 96% of all single-locus genotypes were resolved. The mean number of alleles per locus was 6.9 (range: 3–13) and mean observed heterozygosity was 0.76 (range: 0.58–0.93). We estimated genotyping error rates by re-extracting, amplifying, and genotyping nine individuals and observed complete concordance between duplicate multi-locus genotypes in all cases.

We used standard likelihood approaches implemented in program Cervus v3.0.3 [30], [31] to assign paternity for the 21 juveniles that were sampled with their assumed biological mothers. We included all genotyped subadult (*n* = 4) and adult males (*n* = 18) as potential fathers to account for the fact some individuals that we classified as subadults based on size may have been old enough to breed. Parentage likelihoods were estimated jointly for mothers and putative fathers using a “trio” analysis [30].We assumed a genotyping error rate of 0.01 to take into account the fact that some genotyping errors could have occurred despite the concordance among duplicated genotypes. We set the proportion of sampled candidate fathers at 85% based on the fact that almost all males encountered in our study area have already been captured and sampled. We used “likelihood of the difference” scores, which represent the difference in the likelihood of the two most likely candidate fathers, and a strict confidence level of 95% to assign paternity.

### Pairwise Relatedness and Geographic Distance

We estimated relatedness between all pairs of individuals using ML-Relate [32]. We related pairwise relatedness estimates to the geographic distance (m) separating the original capture locations for members of the pair. We conducted separate analyses for male-male, female-male, and female-female pairs, and limited our analysis to adults because of a small sample size of subadults because subadults may still have been in the process of dispersing to their first breeding site when sampled. To test for relationships between pairwise relatedness and distance between individuals, we used analysis of covariance (ANCOVA) models with relatedness as the dependent variable and distance as an independent variable. In each analysis, we treated distance as both a continuous and categorical (in bins of 500 m) variable to determine if relatedness peaked at a particular distance, which could be indicative of natal dispersal distance.

## Results

In total, we captured 74 sloths: 40 adults (19 males and 21 females), 9 subadults, and 25 juveniles. Adult sloths did not exhibit sexual size dimorphism (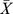 = 4.2 kg, SE = 0.05; *t*_32_ = 0.72, *p* = 0.24), after excluding pregnant females. We obtained of tissue from the leg of 66 captured individuals (we did not collect tissue from 4 juveniles, 2 adult females, 1 subadult female and 1 adult male) for genetic analysis. All mothers shared at least one allele with their putative offspring, confirming the maternity of females sampled with young, with one exception. This mother did not share an allele with either of her two offspring at the same locus, and we suspect that the mismatch was due to a null allele in the female (no other females shared an allele at all loci with either of these two juveniles). We assigned paternity to 20 of the 21 genotyped juveniles with >95% confidence. In all cases, the assigned father shared an allele at all loci with the juvenile in question.

**Figure 2:**
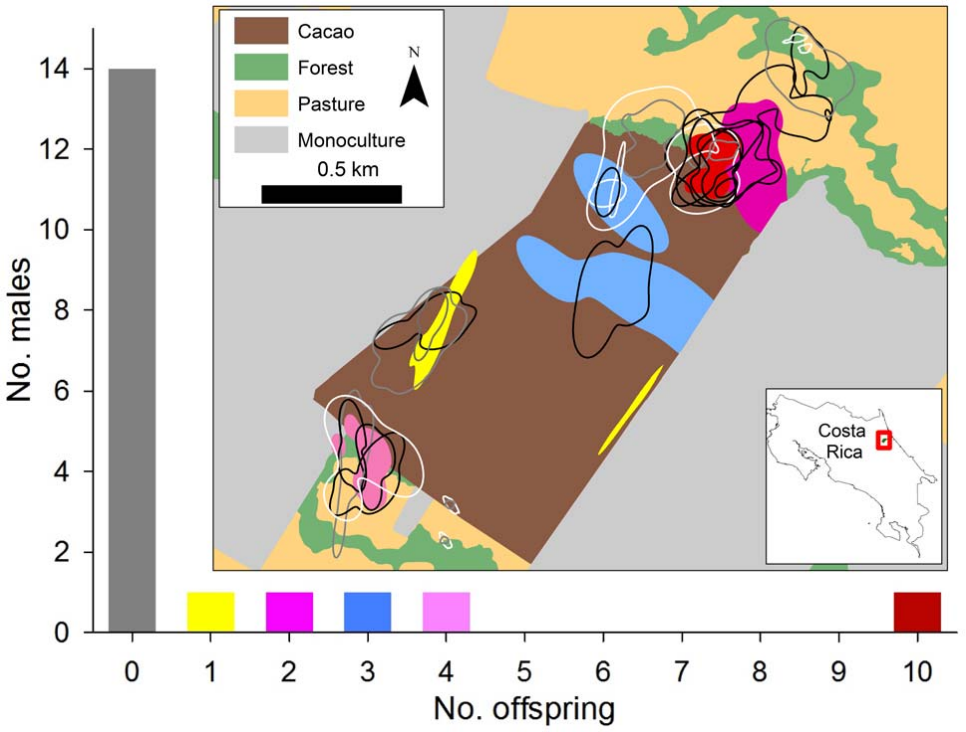
Distribution of reproductive success for adult male (*n* = 20) brown-throated three-toed sloths (*Bradypus variegatus*), and home ranges (90% fixed kernel) of all adult sloths across the four dominant cover types in northeastern Costa Rica, 2010–2012. Males that were assigned paternity to juveniles are represented with colours. Black outlines represent the home ranges of females that were reproductively active. White and grey lines represent adult females and males respectively that did not sire offspring during our study. The home range of one female that dispersed north and out of the study area is depicted prior to her dispersal.

Paternity analyses indicated high levels of reproductive skew among males (Fig. 2). Indeed, paternity for 10 of the 20 juveniles (50%) was assigned to a single male, and paternity for the remaining 10 juveniles was assigned to four adult males. These four males sired one, two, three, and four offspring each. Thus, 14 of 19 (74%) sampled males were not assigned paternity to a single juvenile. On the other hand, reproductive skew for females was low; of the 20 adult females genotyped, six (30%) did not reproduce, eight (40%) produced one offspring, four (20%) had two offspring and two (10%) produced three offspring. Of the two mothers sampled with three different young, one produced offspring exclusively with a single male, and the other produced its offspring with two different males. Of the four mothers sampled with two different young, one produced young with multiple males, and the other three produced young exclusively with a single male.

**Figure 3:**
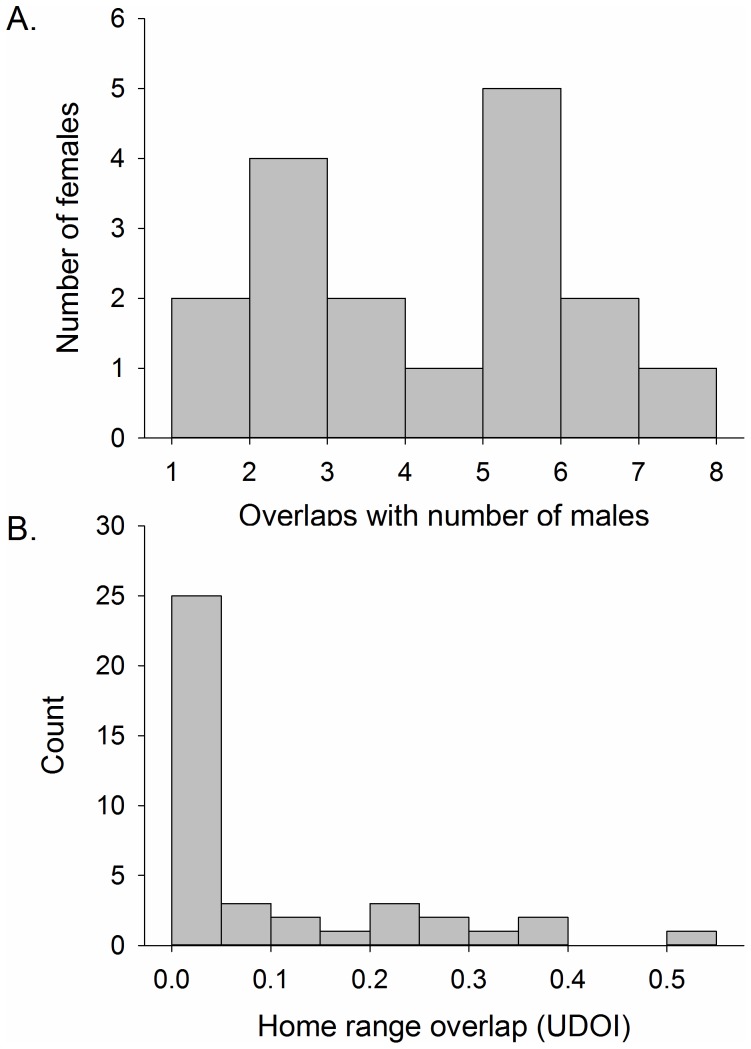
Frequency distribution of A. the number of adult male home ranges in which females overlapped or occurred within and; B. a pairwise index of home range overlap (utilization distribution overlap index or UDOI) for adult male brown-throated three-toed sloths in northeastern Costa Rica, 2010–2012.

Even though the home ranges of an adult intersected an average of four other adults (range 0 to 11; Fig. 2), and adult males overlapped with an average of three males (range 0–8; Fig. 2), the majority of adult males maintained exclusive use of the core of their home range. Indeed, the average amount home range overlap (UDOI = 0.10, SE = 0.01) between pairs of males whose home ranges overlapped was very low (Fig. 3A) and only one male pair exhibited a UDOI >0.5 (Fig. 3A). Moreover, the average home range overlap was 2× higher between males and females (UDOI = 0.20, SE = 0.02) and >4× higher between females and females (UDOI = 0.42, SE = 0.03) than it was for male-male pairs, and these differences were statistically significant (ANOVA *F*_2,171_ = 12.9, *p*<0.001). Most female home ranges intersected the home ranges of multiple males; indeed the average female overlapped with the home range of four males (range 1–7) and all occurred within at least one male’s home range (Fig. 3B). Home range size was variable among both sexes, ranging from 0.25 to 14.8 ha (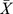 = 4.7 ha, SE = 1.0, *n* = 15) and 0.13 to 19.9 (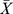 = 5.4 ha, SE = 1.4, *n* = 15) for females and males, respectively (Fig. 2). Home range size did not differ between sexes (*t*_28_ = 0.40, *p* = 0.35) or by breeding status (*t*_28_ = 1.24, *p* = 0.11).

**Figure 4:**
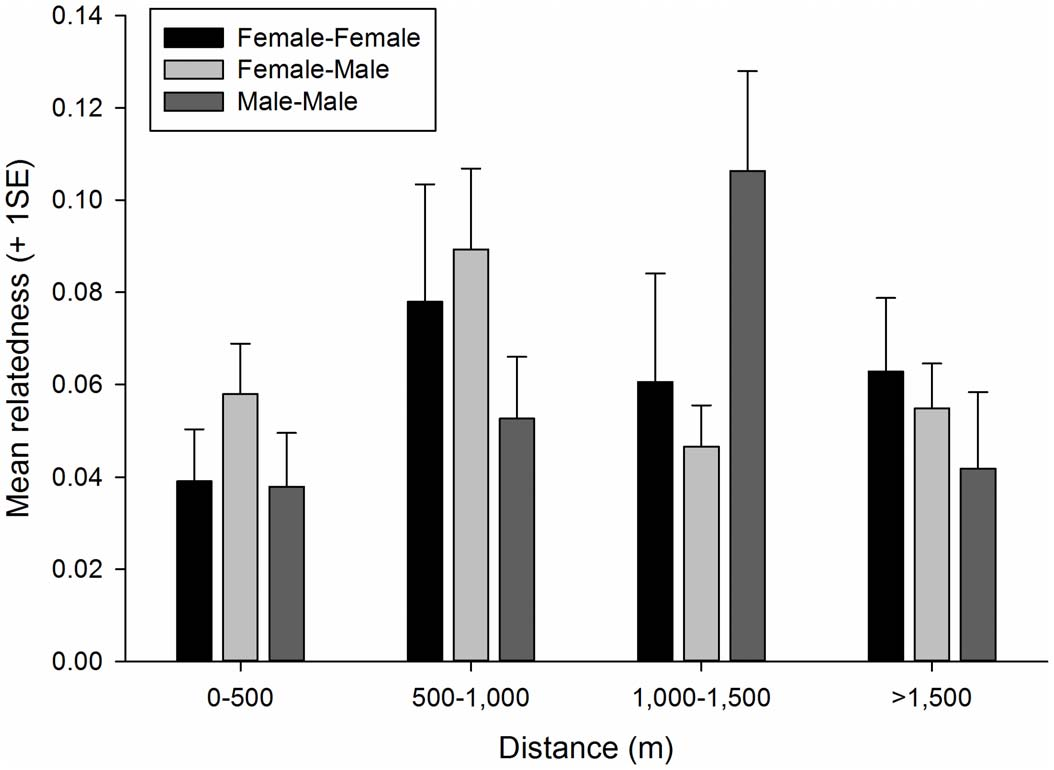
Mean relatedness coefficients for pairs of brown-throated three-toed sloths as a function of geographic distance between pair members in northeastern Costa Rica, 2010–2012.

Relatedness did not decline linearly with increasing geographic distance between pairs of individuals for any of the three gender combinations (*p*-values = 0.45 to 0.75). Pairwise relatedness varied significantly among distance categories for male-male pairs (*F*_7,147_ = 3.61, *p* = 0.02), largely because relatedness was relatively high in the 1,000 to 1,500 m category (Fig. 4). Pairwise relatedness did not differ among distance categories for female-female pairs (*F*_3,166_ = 0.7, *p* = 0.56) or male-female pairs (*F*_3,336_ = 2.0, *p* = 0.12).

## Discussion

Contrary to our predictions, brown-throated three-toed sloths appeared to possess a polygynous mating system characterized by high male reproductive skew. Indeed, 16% of adult males (3/19 individuals) sired 85% of the juveniles (17/20 individuals), and nearly three-quarters (14/19 individuals) of adult males did not sire offspring during our two year study. The observed level of male reproductive skew was not an artefact of high rates of mortality as all candidate fathers survived the duration of the study. Nor was skew the consequence of inadequate sampling as all but one juvenile was assigned to a father with >95% confidence. Thus, the observed level of reproductive skew in three-toed sloths reflects real variation in male mating success and is comparable to that observed in other strongly polygynous mammals including pinnipeds [33] and ungulates [34].

In their classic paper, Emlen and Oring [15] suggested that polygny would be selected against when limiting resources result in widely dispersed females, and individual males are physically incapable of monopolizing females. In other words, when the “polygny potential” of the environment is low, promiscuity or other tactics should be favoured. Consistent with this hypothesis, Beasley et al. [13] detected high rates of promiscuity in Virginia opossums (*Didelphis virginiana*) inhabiting a fragmented landscape. We predicted a similar mating strategy would emerge in three-toed sloths because sloths were limited to fragmented strips of forest, and possessed such limited mobility that males would be unable to monopolize multiple females. However, in contrast to opossums which did not possesses discrete home ranges, female three-toed sloths seemed capable of meeting their life-history requirements within small clearly defined areas (average home range size = ∼5 ha). Small home ranges, coupled with high levels of overlap in the use of space by females allowed some males to, indirectly or directly, control access to multiple females despite their limited mobility and the fragmented habitat.

We consider it unlikely that contest competition among males was the only mechanism behind the observed reproductive skew. Similar to the Atlantic forest maned sloth [24], we did not detect strong sexual size dimorphism in brown-throated sloths, which is often the case for species that guard and compete for access to females [35]. Rather, high male reproductive skew could either be due to females selectively mating with a small number of males or through resource-defence polygny, where males control access to habitats used by females. Certainly, female sloths had multiple mating choices available to them given the average female home range overlapped the home range of almost four males. However, it is also possible that males excluded competitors from core areas given that mean UDOI for overlapping males was minimal and only one pair of males possessed a UDOI >0.5. Moreover, territorial disputes have been observed between male brown-throated three-toed sloths [36]. Thus, both processes may be important, where males that sequester premium habitat attract more females, and have increased breeding opportunities because they are most attractive from the perspective of the females. Our data support this hypothesis; the male (depicted in red in Fig. 2) that sired half of the sampled juveniles held an area intact riparian forest that featured the greatest density of females.

The benefits of sequestering high quality habitats with large numbers of females may be enhanced by the low level of relatedness between pairs of males occurring in close proximity to one another. In contrast, Peery and Pauli [14] observed low levels of reproductive skew among male Hoffman’s two-toed sloth (no male sired more than two offspring), and pairs of adult males in close proximity were more closely related than males separated by greater distances. Peery and Pauli [14] suggested that relatedness could promote a certain level of tolerance among neighbouring males for access to females and lead to kin selection in two-toed sloths. Perhaps, natal dispersal distances in male three-toed sloths exceed those of two-toed sloths which could lead greater intolerance among males and different male reproductive strategies.

Even though we observed strong reproductive skew and polygyny, the mating system of three-toed sloths was not necessarily devoid of promiscuity. Typically, mating systems are defined as promiscuous when females mate with multiple males within a single breeding season. We were unable to determine if females mated with multiple males within breeding seasons using genetic methods since three-toed sloths only produce a one young per breeding event and we did not observe copulation events. However, we detected one instance in which a female produced an offspring with a male other than one of the males whose home range she overlapped. We also detected two instances in which females mated with different males in consecutive breeding seasons. Although not definitive proof for promiscuity, these instances suggest that females do not form long-term, exclusive pair bonds with males, and that males exhibit little mate guarding.

The strong reproductive skew we observed across multiple seasons and the spatial segregation of males were indicative of a primarily polygynous mating system with limited promiscuity. Avoidance of infanticide via paternity confusion has been proposed to the primary reason for promiscuity in female mammals [6]. However, infanticide is unlikely to be an important factor shaping female mating strategies in three-toed sloths because it seems implausible that male three-toed sloths are capable of removing a baby from a mother or in investing the energy and time in pursuing a juvenile. Similarly, alternative selective pressures for promiscuity such as increased litter size and sexual coercion are unlikely to be important in three-toed sloths given that females are constrained to a single offspring and males are unlikely to be able to coerce females into copulation. Nevertheless, our results suggest that the fitness benefits of mating with a single male outweighed the potential benefits associated with promiscuity.

Constructing predictions for mating systems has proven difficult. Indeed, despite the limited movement capacity of sloths and the fragmented landscape in which this population occurred – both of which have been theorized to reduce the polygyny potential of a system [15] – we *a priori* predicted a high level of promiscuity. Surprisingly, we found evidence for a strongly polygynous mating system featuring large male reproductive skew and little promiscuity. The hypothesis that limiting resources drive the distribution of females, which in turn dictates whether males will monopolize breeding opportunities and, thus, determines male mating strategies, is intuitively appealing. However, very few empirical studies demonstrating this process exist (but see Davies and Lundberg [37] for an example). Our observations for three-toed sloths do not support the polygyny potential hypothesis. Instead, our study illustrates the complexity of mating systems and the inherent difficulty of predicting mating systems *a priori* because of the suite of drivers including habitat quality, foraging ecology, vagility, reproductive cycles, and male territoriality that shape vertebrate mating systems.
